# Dr. Charles O. Green

**Published:** 1911-09

**Authors:** 


Dr. Charles O. Green, died at his home in Hornell, N. Y., on August 3, as the result of injuries received in an automobile accident. Dr. Green was alighting from a street car when he was struck by the automobile. He was 51 years old. He had served nearly 20 years as a medical officer in the state militia. At the time of his death he was Captain of the Third Regiment. Dr. Green was health officer of Hornell for many years.

